# Comparison of Sequential Intravesical Gemcitabine and Docetaxel vs Bacillus Calmette-Guérin for the Treatment of Patients With High-Risk Non–Muscle-Invasive Bladder Cancer

**DOI:** 10.1001/jamanetworkopen.2023.0849

**Published:** 2023-02-28

**Authors:** Ian M. McElree, Ryan L. Steinberg, Sarah L. Mott, Michael A. O’Donnell, Vignesh T. Packiam

**Affiliations:** 1 Carver College of Medicine, University of Iowa, Iowa City; 2 Department of Urology, University of Iowa, Iowa City; 3 Holden Comprehensive Cancer Center, University of Iowa, Iowa City

## Abstract

**Importance:**

Due to the ongoing bacillus Calmette-Guérin (BCG) shortage, sequential intravesical gemcitabine and docetaxel has been increasingly used as first-line therapy for high-risk non–muscle-invasive bladder cancer (NMIBC). However, data directly comparing these 2 therapies are lacking.

**Objective:**

To compare the outcomes of patients with high-risk NMIBC treated with gemcitabine and docetaxel vs BCG.

**Design, Setting, and Participants:**

This retrospective cohort study was conducted from January 1, 2011, to December 31, 2021. The median (IQR) duration of follow-up was 23 (12-33) months for patients receiving gemcitabine and docetaxel and 49 (27-79) months for patients receiving BCG. All patients were treated at the University of Iowa tertiary care center. A total of 312 patients with high-risk treatment-naive NMIBC were included; 174 patients were treated with BCG therapy and 138 were treated with gemcitabine and docetaxel therapy.

**Exposures:**

After undergoing complete transurethral resection of bladder tumor, patients received either sequential intravesical gemcitabine, 1 g, and docetaxel, 37.5 mg, or 1 vial of BCG. Induction treatments were administered once per week for 6 weeks. Maintenance regimens were initiated if the patient was disease free at the first follow-up visit.

**Main Outcomes and Measures:**

The primary outcome was high-grade recurrence-free survival (RFS). Survival probabilities were estimated using the Kaplan-Meier method. Cox regression models were used to evaluate the association of covariates with outcomes. Adverse events were reported using the Common Terminology Criteria for Adverse Events, version 5.

**Results:**

Among 312 patients, the median (IQR) age was 73 (66-79) years; 255 patients (81.7%) were male and 292 (93.6%) were White. Baseline clinicopathological characteristics such as sex, smoking status, and pretreatment tumor pathology were similar between treatment groups. High-grade RFS estimates were 76% (95% CI, 69%-82%) at 6 months, 71% (95% CI, 64%-78%) at 12 months, and 69% (95% CI, 62%-76%) at 24 months in the BCG group and 92% (95% CI, 86%-95%) at 6 months, 85% (95% CI, 78%-91%) at 12 months, and 81% (95% CI, 72%-87%) at 24 months in the gemcitabine and docetaxel group. Multivariable Cox regression analyses controlled for age, sex, treatment year, and presence of carcinoma in situ revealed that treatment with gemcitabine and docetaxel was associated with better high-grade RFS (hazard ratio, 0.57; 95% CI, 0.33-0.97; *P* = .04) and RFS (hazard ratio, 0.56; 95% CI, 0.34-0.92; *P* = .02) than treatment with BCG. Induction therapy for BCG was associated with greater treatment discontinuation than induction therapy for gemcitabine and docetaxel (9.2% vs 2.9%; *P* = .02).

**Conclusions and Relevance:**

In this cohort study, gemcitabine and docetaxel therapy was associated with less high-grade disease recurrence and treatment discontinuation than BCG therapy. These findings suggest that, while awaiting results from an ongoing randomized clinical trial during the current BCG shortage, use of gemcitabine and docetaxel can be considered for recommendation in updated practice guidelines.

## Key Points

**Question:**

Is there a difference in outcomes associated with sequential intravesical gemcitabine and docetaxel therapy vs bacillus Calmette-Guérin (BCG) therapy, the standard of care for the treatment of high-risk non–muscle-invasive bladder cancer (NMIBC)?

**Findings:**

In this cohort study of 312 patients with high-risk NMIBC, those receiving gemcitabine and docetaxel had better recurrence-free survival and a lower rate of induction treatment discontinuation than those receiving BCG.

**Meaning:**

These findings suggest that gemcitabine and docetaxel may serve as a reasonable alternative first-line therapy for patients with high-risk NMIBC during the ongoing BCG shortage.

## Teaser

This cohort study compares outcomes of treatment with sequential intravesical gemcitabine and docetaxel vs bacillus Calmette-Guérin among patients with high-risk non–muscle-invasive bladder cancer.

## Introduction

There will be an estimated 81 180 bladder cancer diagnoses in the US in 2022, with approximately 75% being non–muscle-invasive bladder cancer (NMIBC).^1,2^ Since its approval by the Food and Drug Administration in 1990 as the first cancer immunotherapy, bacillus Calmette-Guérin (BCG) has been the gold standard adjuvant therapy for high-risk NMIBC after transurethral resection of bladder tumor (TURBT).^2^ When tested against adjuvant single-agent chemotherapy regimens such as mitomycin and doxorubicin, BCG has had superior prevention of recurrence and progression.^3,4,5,6^ However, BCG is not without its limitations; patients can experience recurrence at a rate of up to 40% at 2 years, while other vulnerable populations, such as older adults and immunocompromised individuals, may experience diminished immunologic response.^7,8,9^ The immunologic nature of BCG is responsible for a variety of potentially severe adverse effects (AEs).^10,11^ However, the most important problem with BCG is that continual production shortages have left many urological practices in need of an effective and readily available alternative first-line treatment.^12,13^ Continued production and standardization issues have created widespread problems for the care of patients with NMIBC.^13,14,15,16^

Sequential intravesical gemcitabine and docetaxel was initially described in 2015 as an effective and well-tolerated therapy after BCG failure, yielding 2-year recurrence-free survival (RFS) of 34% to 46%.^17,18^ In light of promising preliminary results and ongoing BCG production shortages, over the last decade, our institution transitioned to use of gemcitabine and docetaxel as the preferred first-line treatment for NMIBC. A recent retrospective review^19^ of 107 patients with BCG-naive high-risk NMIBC who were treated with gemcitabine and docetaxel at our institution found favorable results for this regimen, including 82% RFS, 84% high-grade RFS, and no local stage or metastatic progression at 2 years. Furthermore, gemcitabine and docetaxel therapy was well tolerated, and only 4% of patients had to discontinue induction treatment.^19^ While these data are promising, there are no studies directly comparing the benefits and tolerability of gemcitabine and docetaxel therapy with those of BCG therapy.^14^ Randomized clinical trials often take many years to complete and, during the current period of BCG shortage, the lack of a well-studied alternative treatment has come at the cost of patient outcomes.^20,21^ Therefore, to fill this gap, we aimed to compare oncological outcomes between patients treated with BCG vs gemcitabine and docetaxel for high-risk treatment-naive NMIBC at our institution between 2011 and 2021.

## Methods

### Study Design and Population

After obtaining approval from the University of Iowa Institutional Review Board, we retrospectively reviewed all patients with pathologically confirmed high-risk NMIBC who were treated with either gemcitabine and docetaxel or BCG as first-line therapy at the University of Iowa tertiary care center between January 1, 2011, and December 31, 2021. A waiver of informed consent was granted due to the use of deidentified data. All patients were treatment naive at the start of the study and had no history of high-grade bladder cancer. Patient race and ethnicity were self-reported and abstracted from the electronic health record; these characteristics were collected because they have been associated with outcome disparities in urological cancer. Risk stratification was performed per American Urological Association criteria.^2^ A complete induction course was defined as 5 of 6 instillations for both treatment regimens. Patients were excluded if they did not undergo follow-up surveillance during the study period. After exclusions, the final cohort included 312 patients with high-risk NMIBC. This study followed the Strengthening the Reporting of Observational Studies in Epidemiology (STROBE) reporting guideline for cohort studies.

### BCG Therapy

The BCG treatment protocol has been previously reported.^22^ In brief, patients received 1 vial of BCG TICE strain (Organon Teknika Corporation) with or without 50 million U of interferon α-2b (IFN-α-2b) instilled simultaneously into the bladder for approximately 90 to 120 minutes. The starting BCG dose of the induction cycle was altered to one-third of the dose during times when BCG availability was limited at our institution or when a patient experienced substantial lower urinary tract symptoms before treatment. The BCG maintenance regimen consisted of a reduced (one-third to one-tenth) dose administered over three 3-week courses at 3 months, 9 months, and 15 months after the end of the last induction treatment, as previously described.^22^

### Gemcitabine and Docetaxel Therapy

The gemcitabine and docetaxel treatment protocol has been previously reported.^17^ In brief, patients received 1 g of gemcitabine in 50 mL of sterile water or normal saline instilled into the bladder for approximately 90 minutes, followed by 37.5 mg of docetaxel in 50 mL of saline for approximately 90 to 120 minutes. Induction treatments took place once per week for 6 weeks. Oral ondansetron, 4 mg, and naproxen, 250 mg, were provided if nausea or bladder pain developed. Prophylactic oxybutynin, 5 mg, was given to patients with known bladder spasms. If disease free at the first surveillance visit, monthly maintenance therapy was initiated for up to 24 months; this maintenance therapy replicated the procedures and doses used during induction therapy.

### Surveillance

Surveillance was performed 4 to 8 weeks after completion of therapy induction and involved formal restaging or an office cystoscopy. Formal restaging procedures included cystoscopy (with blue light in most cases), bladder barbotage cytological testing, bilateral upper-tract barbotage cytological testing, bilateral retrograde pyelogram, random bladder biopsy, and prostatic urethral biopsy. Patients with negative cystoscopic results and suspicious or positive cytological results during surveillance underwent additional restaging procedures. Patients with low-grade recurrences received repeat TURBT or office fulguration and were considered for continued maintenance or reinduction therapy. When patients were disease free, repeat cystoscopic and bladder cytological testing was performed quarterly for 2 years, twice annually for 2 more years, and annually thereafter. Routine upper-tract imaging was obtained every 1 to 2 years until year 5, then as indicated.

### Statistical Analysis

After institutional review board approval, patient medical records were retrospectively reviewed, and data were stored in a Research Electronic Data Capture (REDCap) database supported by the University of Iowa (via funding from the National Institutes of Health/Clinical and Translational Science Awards program). Patients’ clinicopathological features, treatment history, tolerance to therapy, and oncological outcomes were analyzed. The primary outcome was high-grade recurrence-free survival (RFS). Secondary outcomes included RFS, progression-free survival (PFS), cystectomy-free survival (CFS), cancer-specific survival (CSS), and overall survival (OS). Adverse events were classified using the National Cancer Institute Common Terminology Criteria for Adverse Events (CTCAE), version 5. When symptoms had a unifying cause (eg, lower urinary tract symptoms during urinary tract infection), only the latter unifying CTCAE term was reported. Intolerance was defined as an inability to continue treatment due to the development of AEs.

We used χ^2^ tests to compare categorical variables and *t* tests to compare continuous variables (baseline characteristics and AEs) between treatment groups. Survival probabilities were estimated and plotted using the Kaplan-Meier method. Estimates along with pointwise 95% CIs were reported.

Recurrence-free survival was defined as time from the start of therapy induction to recurrence, and high-grade RFS was defined as time from the start of therapy induction to high-grade recurrence, respectively. Recurrence was defined as tumor relapse in the bladder or prostatic urethra (for male patients). Progression-free survival was defined as time from the start of therapy induction to progression, with progression defined as the development of a T2 or greater disease, lymph node or metastatic disease, or receipt of any cystectomy. Otherwise, patients were censored at the last urological follow-up visit. Cancer-specific survival was defined as time from the start of therapy induction to death due to bladder cancer. Patients were censored at the date of death due to other causes or at the date they were last known to be alive. Overall survival was defined as time from the start of therapy induction to death due to any cause. Duration of response was defined as time from initial surveillance to recurrence (high or low grade) among those who hadn’t experienced recurrence at the initial surveillance visit. Patients still alive were censored at the last known follow-up visit.

Cox regression models were used to evaluate the associations of patient, disease, and treatment characteristics with RFS and high-grade RFS. All statistical testing was 2-sided, with *P* = .05 set as the significance threshold. Data were analyzed using SAS software, version 9.4 (SAS Institute Inc).

## Results

### Patient Demographic Characteristics

Among 312 patients with high-grade NMIBC (median [IQR] age, 73 [66-79] years; 255 [91.7%] male, 57 [18.3%] female, and 292 [93.6%]) White); 174 received BCG and 138 received gemcitabine and docetaxel (Table 1). The BCG vs gemcitabine and docetaxel groups had similar clinicopathological characteristics; 139 (79.9%) vs 116 (84.1%) were male, 35 (20.1%) vs 22 (15.9%) were female, 122 (70.1%) vs 90 (65.2%) had a history of any smoking, 76 (43.7%) vs 56 (40.6%) had any pretreatment carcinoma in situ (CIS), and 102 (58.6%) vs 76 (55.1%) had any pretreatment T1 disease. However, patients receiving gemcitabine and docetaxel were older than those receiving BCG (median [IQR] age, 74 [67-82] years vs 71 [64-77] years; *P* = .002). In addition, due to temporal changes in BCG availability and our institutional protocol, patients were more likely to receive gemcitabine and docetaxel than BCG after 2019 (107 patients [77.5%] vs 41 patients [23.6%]; *P* < .001). Within the BCG group, 137 patients (78.7%) received IFN-α-2b concurrently with BCG during intravesical treatments. Blue light cystoscopy was used at the first 3-month surveillance visit in 131 of 161 eligible patients (81.4%) in the BCG group and 117 of 136 eligible patients (86.0%) in the gemcitabine and docetaxel group. Most eligible patients in both groups received maintenance therapy (109 patients [94.0%] in the BCG group and 112 patients [89.6%] in the gemcitabine and docetaxel group).

**Table 1.** Clinicopathological Characteristics of Patients

Characteristic	Patients, No./total No. (%)		*P* value
	BCG group (n = 174)	Gemcitabine and docetaxel group (n = 138)	
Age, median (IQR)	71 (64-77)	74 (67-82)	.002
Sex			
Female	35/174 (20.1)	22/138 (15.9)	.34
Male	139/174 (79.9)	116/138 (84.1)	
Race and ethnicity^a^			
White	163/172 (94.8)	129/138 (93.5)	.63
Other^b^	9/172 (5.2)	9/138 (6.5)	
Smoking status			
Never	52/174 (29.9)	48/138 (34.8)	.08
Current	32/174 (18.4)	13/138 (9.4)	
Former	90/174 (51.7)	77/138 (55.8)	
Tumor size^c^^,^^d^			
Small	12/102 (11.8)	11/71 (15.5)	.35
Medium	37/102 (36.3)	31/71 (43.7)	
Large	53/102 (52.0)	29/71 (40.8)	
Multifocal tumor^c^^,^^e^			
No	67/130 (51.5)	45/97 (46.4)	.44
Yes	63/130 (48.5)	52/97 (53.6)	
Pretreatment tumor pathology			
CIS alone	23/174 (13.2)	17/138 (12.3)	.46
T1	65/174 (37.4)	56/138 (40.6)	
T1 plus CIS	37/174 (21.3)	20/138 (14.5)	
Ta	33/174 (19.0)	26/138 (18.8)	
Ta plus CIS	16/174 (9.2)	19/138 (13.8)	
Pretreatment CIS-containing tumor			
No	98/174 (56.3)	82/138 (59.4)	.58
Yes	76/174 (43.7)	56/138 (40.6)	
Treatment year			
2011-2018	133/174 (76.4)	31/138 (22.5)	<.001
2019-2021	41/174 (23.6)	107/138 (77.5)	

Abbreviations: BCG, bacillus Calmette-Guérin; CIS, carcinoma in situ.

^a^ Data were missing for 2 patients in the BCG group.

^b^ Other self-reported racial and ethnic categories included African American or Black, Asian, Hispanic or Latino, and multiple (≥2) races and/or ethnicities.

^c^ These variables apply to papillary pathology only.

^d^ Data were missing for 49 patients in the BCG group and 50 patients in the gemcitabine and docetaxel group.

^e^ Data were missing for 21 patients in the BCG group and 24 patients in the gemcitabine and docetaxel group.

### Survival Outcomes

Median (IQR) follow-up times for survival end points were 49 (27-79) months for patients receiving BCG and 23 (12-33) months for patients receiving gemcitabine and docetaxel. In the BCG group, 69 patients experienced recurrence, 60 experienced high-grade recurrence, 24 experienced progression, 37 died, and 10 died of cancer-related causes. In the gemcitabine and docetaxel group, 32 patients experienced recurrence, 28 experienced high-grade recurrence, 3 experienced progression, 21 died, and 0 died of cancer-related causes.

For the primary outcome of high-grade RFS, 6-month estimates were 76% (95% CI, 69%-82%) in the BCG group and 92% (95% CI, 86%-95%) in the gemcitabine and docetaxel group, and 1-year estimates were 71% (95% CI, 64%-78%) in the BCG group and 85% (95% CI, 78%-91%) in the gemcitabine and docetaxel group. Two-year estimates were 69% (95% CI, 62%-76%) in the BCG group and 81% (95% CI, 72%-87%) in the gemcitabine and docetaxel group.

For secondary survival outcomes, the 2-year estimates in the BCG group were 62% (95% CI, 54%-69%) for RFS, 94% (95% CI, 88%-97%) for CFS, 92% (95% CI, 86%-95%) for PFS, 98% (95% CI, 94%-99%) for CSS, and 93% (95% CI, 88%-96%) for OS. The 2-year estimates in the gemcitabine and docetaxel group were 78% (95% CI, 69%-85%) for RFS, 98% (95% CI, 93%-100%) for CFS, 97% (95% CI, 92%-99%) for PFS, 100% (95% CI, 100%-100%) for CSS, and 89% (95% CI, 81%-94%) for OS (Table 2).

**Table 2.** Summary of Survival Estimates and Follow-up

Outcome	Estimated survival rate, % (95% CI)				Follow-up, median (IQR) mo^a^
	6 mo	12 mo	24 mo	36 mo	
**RFS**					
BCG	72 (64-78)	67 (60-74)	62 (54-69)	60 (51-67)	43 (19-76)
Gemcitabine and docetaxel	90 (84-94)	83 (75-89)	78 (69-85)	66 (53-77)	20 (10-32)
**High-grade RFS**					
BCG	76 (69-82)	71 (64-78)	69 (62-76)	66 (58-73)	43 (20-75)
Gemcitabine and docetaxel	92 (86-95)	85 (78-91)	81 (72-87)	71 (58-81)	20 (12-32)
**PFS**					
BCG	95 (91-98)	95 (90-97)	92 (86-95)	88 (82-93)	44 (23-75)
Gemcitabine and docetaxel	99 (95-100)	98 (93-100)	97 (92-99)	97 (92-99)	21 (12-34)
**CFS**					
BCG	97 (93-99)	96 (92-98)	94 (88-97)	93 (87-96)	43 (23-72)
Gemcitabine and docetaxel	99 (95-100)	98 (93-100)	98 (93-100)	98 (93-100)	21 (12-34)
**CSS**					
BCG	99 (96-100)	99 (95-100)	98 (94-99)	97 (93-99)	48 (26-78)
Gemcitabine and docetaxel	100 (100-100)	100 (100-100)	100 (100-100)	100 (100-100)	23 (13-34)
**OS**					
BCG	98 (95-99)	96 (91-98)	93 (88-96)	90 (84-94)	49 (27-79)
Gemcitabine and docetaxel	98 (93-99)	98 (93-99)	89 (81-94)	85 (73-91)	23 (12-33)

Abbreviations: BCG, bacillus Calmette-Guérin; CFS, cystectomy-free survival; CSS, cancer-specific survival; OS, overall survival; PFS, progression-free survival; RFS, recurrence-free survival.

^a^ Median follow-up time for patients surviving the outcome of interest.

Gemcitabine and docetaxel therapy was associated with better RFS (eFigure 1 in Supplement 1) and high-grade RFS (Figure) compared with BCG therapy. The 2-year duration of response estimates were 83% in both treatment groups. There were distinct response patterns between agents. During the initial 3- to 6-month period, a lower response level was observed in the BCG group compared with the gemcitabine and docetaxel group. However, the BCG group had a more durable response over time.

**Figure.**
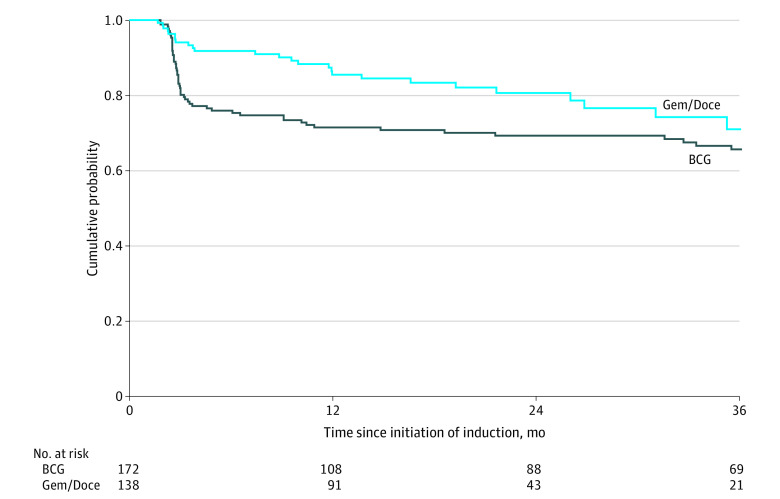
High-grade Recurrence-Free Survival by Treatment Group BCG indicates bacillus Calmette-Guérin; and Gem/Doce, gemcitabine and docetaxel.

There were 27 progression events; of those, 3 occurred in the gemcitabine and docetaxel group and 24 occurred in the BCG group. In the gemcitabine and docetaxel group, 2 patients underwent cystectomy for end-stage bladder cancer symptoms, and 1 patient developed metastatic urothelial cancer. In the BCG group, 6 patients underwent radical cystectomy for recurrent NMIBC, 8 underwent cystectomy after developing muscle-invasive bladder cancer (1 partial cystectomy), 1 developed muscle-invasive bladder cancer but did not pursue radical cystectomy, 1 underwent radical cystectomy for end-stage bladder cancer symptoms, and 8 died of metastatic bladder cancer. Pathological findings after cystectomy are summarized in the eTable in Supplement 1. The PFS, CFS, CSS, and OS estimates were similar between groups (eFigures 2-5 in Supplement 1).

After adjusting for age, sex, treatment year, and presence of CIS, treatment with gemcitabine and docetaxel was associated with a lower risk of recurrence (hazard ratio [HR], 0.56; 95% CI, 0.34-0.92; *P* = .02) compared with treatment with BCG (Table 3). Adjusting for the same factors, gemcitabine and docetaxel therapy was also associated with a lower risk of high-grade recurrence (HR, 0.57; 95% CI, 0.33-0.97; *P* = .04) than BCG therapy. In addition, patients with pretreatment CIS-containing tumors had a greater risk of high-grade recurrence (HR, 1.86; 95% CI, 1.21-2.84; *P* = .005) than patients without CIS-containing tumors.

**Table 3.** Cox Regression Analysis of Risk Factors Associated With Disease Recurrence^a^

Factor	Patients, No.	RFS		High-grade RFS	
		HR (95% CI)	*P* value	HR (95% CI)	*P* value
Age	310	1.11 (0.90-1.37)	.32	1.11 (0.89-1.38)	.37
Sex					
Female	56	0.90 (0.54-1.50)	.68	1.01 (0.59-1.73)	.96
Male	254	1 [Reference]		1 [Reference]	
Treatment year					
2019-2021	147	1.10 (0.68-1.80)	.69	1.20 (0.70-2.04)	.51
2011-2018	163	1 [Reference]		1 [Reference]	
Pretreatment CIS-containing tumor					
Yes	132	1.38 (0.93-2.05)	.11	1.86 (1.21-2.84)	.005
No	178	1 [Reference]		1 [Reference]	
Treatment					
Gemcitabine and docetaxel	138	0.56 (0.34-0.92)	.02	0.57 (0.33-0.97)	.04
BCG	172	1 [Reference]		1 [Reference]	

Abbreviations: BCG, bacillus Calmette-Guérin; CIS, carcinoma in situ; HR, hazard ratio; RFS, recurrence-free survival.

^a^ The number of observations in the original data set was 312, and the number of observations used was 310.

### Tolerance

A summary of AEs is shown in Table 4. A total of 86 patients (49.4%) in the BCG group and 72 patients (52.2%) in the gemcitabine and docetaxel group reported experiencing AEs. Most events were transient and classified as grade 1 or 2. Grade 3 to 5 AEs occurred in 7 patients (4.0%) in the BCG group and 2 patients (1.4%) in the gemcitabine and docetaxel group. Patients who received BCG were more likely to discontinue induction therapy compared with those who received gemcitabine and docetaxel (16 patients [9.2%] vs 4 patients [2.9%]; *P* = .02) (Table 4). Among patients treated with BCG, the most frequently reported grade 1 to 2 AEs were dysuria (22 patients [12.6%%]), urinary urgency or frequency (20 patients [11.5%]), and hematuria (19 patients [10.9%]), and the most common grade 3 to 5 AEs were complicated urinary tract infections (UTIs; 4 patients [2.3%]). Among patients treated with gemcitabine and docetaxel, the most frequently reported grade 1 to 2 AEs were bladder spasms (29 patients [21.0%]), UTIs (12 patients [8.7%]) and urinary urgency or frequency (12 patients [8.7%]), and the most frequently reported grade 3 to 5 AEs were complicated UTIs (1 patient [0.7%]) and syncope (1 patient [0.7%]).

**Table 4.** Adverse Events After Treatment

Outcome^a^	Patients, No. (%)								*P* value
	BCG group (n = 174)				Gemcitabine and docetaxel group (n = 138)				
	No. of events				No. of events				
	1-2	3	4	5	1-2	3	4	5	
Patients reporting AEs	86 (49.4)	0	0	0	72 (52.2)	0	0	0	.63
Intolerance to induction therapy	16 (9.2)	0	0	0	4 (2.9)	0	0	0	.02
Type of AE									
Bladder spasms	9 (5.2)	0	0	0	29 (21.0)	0	0	0	<.001
Urinary urgency or frequency	20 (11.5)	0	0	0	12 (8.7)	0	0	0	.46
Dysuria	22 (12.6)	0	0	0	7 (5.1)	0	0	0	.03
Hematuria	19 (10.9)	0	0	0	8 (5.8)	0	0	0	.16
Urinary tract infection	12 (6.9)	3 (1.7)	0	1 (0.6)	12 (8.7)	1 (0.7)	0	0	.77
Noninfective cystitis	1 (0.6)	0	0	0	0	0	0	0	>.99
Bladder pain	3 (1.7)	0	0	0	3 (2.2)	0	0	0	.77
Flank pain	1 (0.6)	0	0	0	0	0	0	0	>.99
Retention	3 (1.7)	0	0	0	3 (2.2)	0	0	0	>.99
Fatigue and/or flulike symptoms	12 (6.9)	0	0	0	7 (5.1)	0	0	0	.64
Fever	4 (2.3)	0	0	0	0	0	0	0	.13
Arthralgia	7 (4.0)	0	0	0	0	0	0	0	.02
Nausea	1 (0.6)	0	0	0	5 (3.6)	0	0	0	.09
Edema	1 (0.6)	1 (0.6)	0	0	0	0	0	0	>.99
Syncope	0	0	0	0	0	1 (0.7)	0	0	.44
Transient ischemic attack	1 (0.6)	0	0	0	0	0	0	0	>.99
Vertigo	0	1 (0.6)	0	0	0	0	0	0	>.99
Pneumonitis	1 (0.6)	0	0	0	0	0	0	0	>.99
Rash	0	0	0	0	1 (0.7)	0	0	0	.44
Sialadenitis	0	1 (0.6)	0	0	0	0	0	0	>.99

Abbreviations: AE, adverse event; BCG, bacillus Calmette-Guérin.

^a^ When symptoms had a unifying cause (eg, lower urinary tract symptoms during urinary tract infection), only the latter unifying Common Terminology Criteria for Adverse Events term was reported.

Patients treated with gemcitabine and docetaxel vs BCG were more likely to experience bladder spasms (29 patients [21.0%] vs 9 patients [5.2%]; *P* < .001), while those treated with BCG vs gemcitabine and docetaxel were more likely to experience dysuria (22 patients [12.6%] vs 7 patients [5.1%]; *P* = .03) and arthralgias (7 patients [4.0%] vs 0 patients; *P* = .02). Urinary tract symptoms, such as urinary urgency or frequency (20 patients [11.5%] in the BCG group vs 12 patients [8.7%] in the gemcitabine and docetaxel group; *P* = .46), hematuria (19 patients [10.9%] in the BCG group vs 8 patients [5.8%] in the gemcitabine and docetaxel group; *P* = .16), and UTIs (16 patients [9.2%] in the BCG group vs 13 patients [9.4%] in the gemcitabine and docetaxel group; *P* = .77) were common in both groups. Although the differences were not statistically significant, nausea was more common in the gemcitabine and docetaxel group vs the BCG group (5 patients [3.6%] vs 1 patient [0.6%]; *P* = .09), and fatigue and/or flulike symptoms were more common in the BCG group vs the gemcitabine and docetaxel group (12 patients [6.9%] vs 7 patients [5.1%]; *P* = .64). The 1 death that occurred was in the BCG group and was associated with a systemic infection after a UTI.

## Discussion

This cohort study had several key findings. First, the 2 treatment groups had similar clinicopathological characteristics, including sex, smoking status, pretreatment tumor pathology, and presence of CIS. Next, a multivariable analysis revealed that patients treated with gemcitabine and docetaxel had a lower risk of recurrence and high-grade recurrence compared with patients treated with BCG. In terms of toxic effects, gemcitabine and docetaxel was well tolerated, and patients receiving gemcitabine and docetaxel were less likely to discontinue treatment induction due to treatment-related AEs compared with patients receiving BCG.

The results in the BCG group are consistent with benchmark BCG outcomes reported in the literature. Historically, in 2 large North American studies,^7,22^ BCG has had 1-year recurrence rates of approximately 70%, decreasing to approximately 60% at 2 years. Recent studies have found that BCG outcomes appear to have improved, with high-risk cohorts reaching 1-year high-grade RFS rates closer to 80%.^23,24^ The improvements in outcomes over time may be associated with use of blue light cystoscopy, improved resection techniques, standardized risk stratification and surveillance protocols, and increasing focus on reporting high-grade RFS rather than RFS over time. We saw comparable results in the current study; patients at high risk who were treated with BCG had a 1-year high-grade RFS rate of 71% and a 2-year high-grade RFS rate of 69%.

Interestingly, there were notably distinct response patterns between agents, as shown in the Figure. During the initial 3- to 6-month time frame, the BCG group experienced a lower degree of response relative to the gemcitabine and docetaxel group. However, the BCG group maintained response more durably over time, with a flattening of the recurrence curve. We surmise that this response pattern is secondary to the distinct mechanism of action of both agents. Some patients may not be able to mount a sufficient immunologic response to BCG and thus experience early recurrence.^25^ Conversely, in those able to mount an effective immunologic response, a more sustained response is possible.^25^ This interpretation is supported by results from previous studies^26,27^ that found lower benefit of BCG treatment among older adults who may be increasingly immunosenescent. Gemcitabine and docetaxel therapy seems to produce a more reliable initial response, although there is relatively consistent attrition over time.^28^

Treatment tolerability was historically a concern with BCG treatment, although with proper protocols and monitoring, the likelihood of experiencing major AEs from BCG induction therapy remains low.^10^ Nevertheless, irritative lower urinary tract symptoms, such as hematuria, dysuria, urinary urgency or frequency, and UTIs, are common and can make BCG treatment adherence difficult.^10^ Many similar AEs are a concern for gemcitabine and docetaxel therapy, but our results revealed that treatment discontinuation rates were lower for this chemotherapy doublet than for BCG. These findings are consistent with those of Pareek et al,^29^ who found in a prospective cohort of 60 patients that gemcitabine and docetaxel therapy was associated with better quality of life outcomes and fewer toxic effects when compared with BCG therapy. While overall tolerability was similar, patients in the present cohort who received gemcitabine and docetaxel were more likely to experience bladder spasms and nausea, while those who received BCG were more likely to experience distinct immunologic AEs, such as arthralgias and fatigue or flulike symptoms. This difference in AEs likely stems from differing mechanisms of action, as treatment with gemcitabine and docetaxel requires additional dwell time owing to sequential instillations but does not appear to produce substantial systemic immunological AEs.

There are a variety of novel agents and regimens under ongoing evaluation as alternative first-line treatments for high-risk NMIBC. The majority of these regimens involve the use of alternative BCG strains (S1602) or combine BCG with additional immunotherapies, such as durvalumab,^30^ atezolizumab,^31^ sasanlimab,^32^ or N-803.^33^ While these clinical trials are certainly of interest, they cannot alleviate the burden of the BCG shortage completely. Furthermore, although many of the agents suggesting promise in the BCG-unresponsive setting, such as nadofaragene firadenovec and cretostimogene grenadenorepvec, may be beneficial for those with BCG-naive disease, the clear advantages of gemcitabine and docetaxel therapy over these regimens include its low cost (due to these chemotherapy agents being generic) and excellent tolerability profile.^34,35^

The results of the current study provide a compelling rationale to support further research efforts into gemcitabine and docetaxel. First, multi-institutional and prospective validation is needed. Single-arm protocols prospectively evaluating gemcitabine and docetaxel are ongoing.^36^ A cooperative group phase 3 randomized clinical trial of treatment with gemcitabine and docetaxel vs BCG^37^ was recently activated, although results will be pending for several years during accrual and analysis. Next, further research to identify factors associated with response will aid in patient selection for BCG vs alternative treatments, particularly during the BCG shortage. Novel paradigms for sequencing of bladder-sparing therapies will be required when BCG shortage precludes its administration as either a first-line or salvage treatment.^38^

### Limitations

This study has several limitations. The retrospective design allowed for selection bias and confounding. Although reported AEs were assigned according to CTCAE criteria, this process was reliant on variable AE reporting standards throughout the study period. While differences in baseline characteristics, such as age and year of treatment, were controlled for using Cox regression multivariable analysis, unmeasured residual confounding could exist. There was a substantial difference in follow-up time between the groups due to the increasing use of gemcitabine and docetaxel since 2019. Longer follow-up and larger sample size could have yielded differences in oncological outcomes such as PFS and OS. Treatment with BCG was administered using our institutional protocol, with patients sometimes receiving one-third dosing in combination with IFN-α-2b; however, neither of these changes in treatment protocol has been associated with adverse oncological outcomes compared with the strict 3-year full-dose Southwest Oncology Group maintenance regimen.^7,22,39^ The results of the present study are derived from patient data from a large-volume single institution with rigorous NMIBC protocols and may not be representative of practices in lower-volume centers.

## Conclusions

In this single-center retrospective cohort study of 312 patients treated over a single decade, patients treated with adjuvant gemcitabine and docetaxel for high-risk NMIBC after TURBT had improved high-grade RFS and less treatment discontinuation compared with those treated with BCG. While awaiting results from an ongoing randomized clinical trial and in the setting of the current BCG shortage, the findings of this study support the use of gemcitabine and docetaxel for the treatment of high-risk NMIBC, suggesting that this regimen can be considered for recommendation in updated practice guidelines. Further prospective evaluation is needed.

## Notes

Supplement 1. **eFigure 1.** Recurrence-Free Survival by Treatment Group **eTable.** Clinical and Pathological Features of Patients Receiving Cystectomy **eFigure 2.** Progression-Free Survival by Treatment Group **eFigure 3.** Cystectomy-Free Survival by Treatment Group **eFigure 4.** Cancer-Specific Survival by Treatment Group **eFigure 5.** Overall Survival by Treatment Group

Supplement 2.
Data Sharing Statement
