# Supplementary material for: Comparison of Sequential Intravesical Gemcitabine and Docetaxel vs Bacillus Calmette-Guérin for the Treatment of Patients With High-Risk Non–Muscle-Invasive Bladder Cancer
Source: JAMA Netw Open. 2023 Feb 28;6(2):e230849. doi: 10.1001/jamanetworkopen.2023.0849 (PMC9975907; doi:10.1001/jamanetworkopen.2023.0849)
Supplement: Supplement 1. — eFigure 1. Recurrence-Free Survival by Treatment Group eTable. Clinical and Pathological Features of Patients Receiving Cystectomy eFigure 2. Progression-Free Survival by Treatment Group eFigure 3. Cystectomy-Free Survival by Treatment Group eFigure 4. Cancer-Specific Survival by Treatment Group eFigure 5. Overall Survival by Treatment Group [file jamanetwopen-e230849-s001.pdf]

Supplementary Online Content

McElree IM, Steinberg RL, Mott SL, O'Donnell MA, Packiam VT. Comparison of sequential intravesical gemcitabine and docetaxel vs bacillus Calmette-Guérin for the treatment of patients with high-risk non–muscle-invasive bladder cancer. *JAMA Netw Open*. 2023;6(2):e230849. doi:10.1001/jamanetworkopen.2023.0849

eFigure 1. Recurrence-Free Survival by Treatment Group

eTable. Clinical and Pathological Features of Patients Receiving Cystectomy

eFigure 2. Progression-Free Survival by Treatment Group

eFigure 3. Cystectomy-Free Survival by Treatment Group

eFigure 4. Cancer-Specific Survival by Treatment Group

eFigure 5. Overall Survival by Treatment Group

This supplemental material has been provided by the authors to give readers additional information about their work.

© 2023 McElree IM et al. *JAMA Network Open*.

eFigure 1. Recurrence-Free Survival by Treatment Group

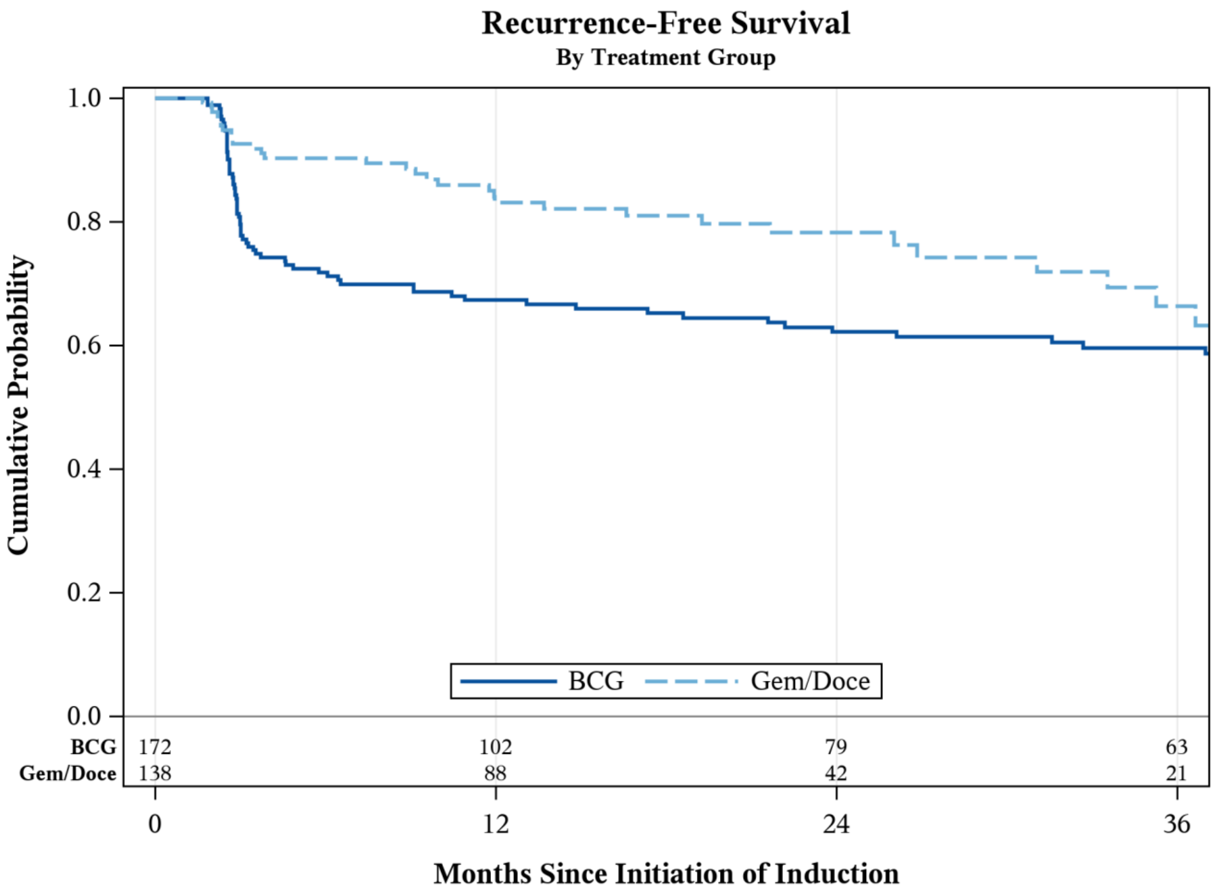

© 2023 McElree IM et al. *JAMA Network Open*.

eTable. Clinical and Pathological Features of Patients Receiving Cystectomy

Treatment Group	Presenting Pathology	Cystectomy Pathology
BCG	T1HG + CIS	pT0N0MX
BCG	T1HG	pT1N1MX
BCG	T1HG + CIS	pT4N0MX
BCG	T1HG + CIS	pT0N0MX
BCG	CIS	pT3aN0MX
BCG	T1HG + CIS	pTisN0MX
BCG	T1HG	pT0N0MX
BCG	T1HG + CIS	pT0N0MX
BCG	TaLG + CIS	pT2N0MX
BCG	TaHG	pT1N0MX
BCG	CIS	pT1N0MX
BCG	T1HG + CIS	pT1N0MX
BCG	T1HG + CIS	pT1N0MX
BCG	TaHG	pT3bN0MX
BCG	TaHG	pTisN0MX
Gem/Doce	T1HG + CIS	pTisN0MX
Gem/Doce	TaHG + CIS	pTisN0MX

© 2023 McElree IM et al. *JAMA Network Open*.

eFigure 2. Progression-Free Survival by Treatment Group

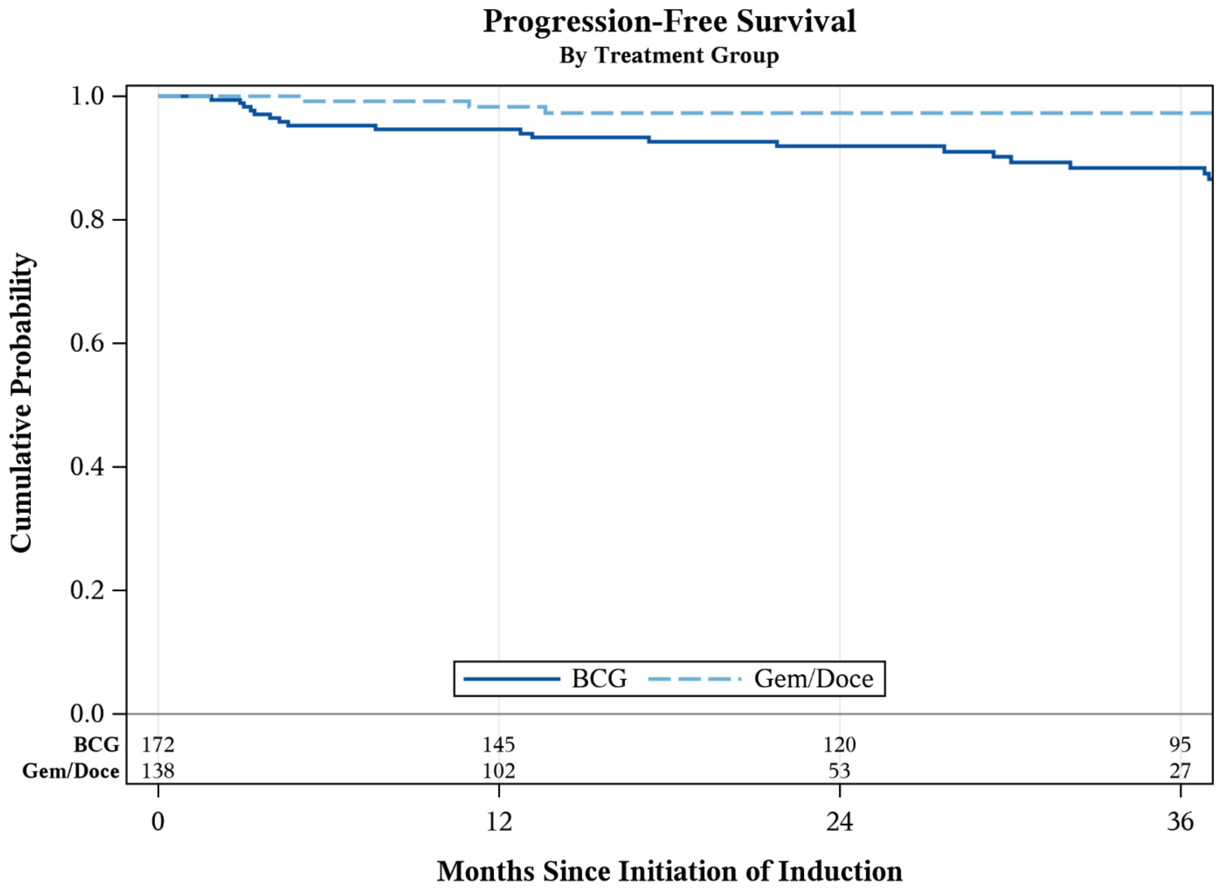

© 2023 McElree IM et al. *JAMA Network Open*.

eFigure 3. Cystectomy-Free Survival by Treatment Group

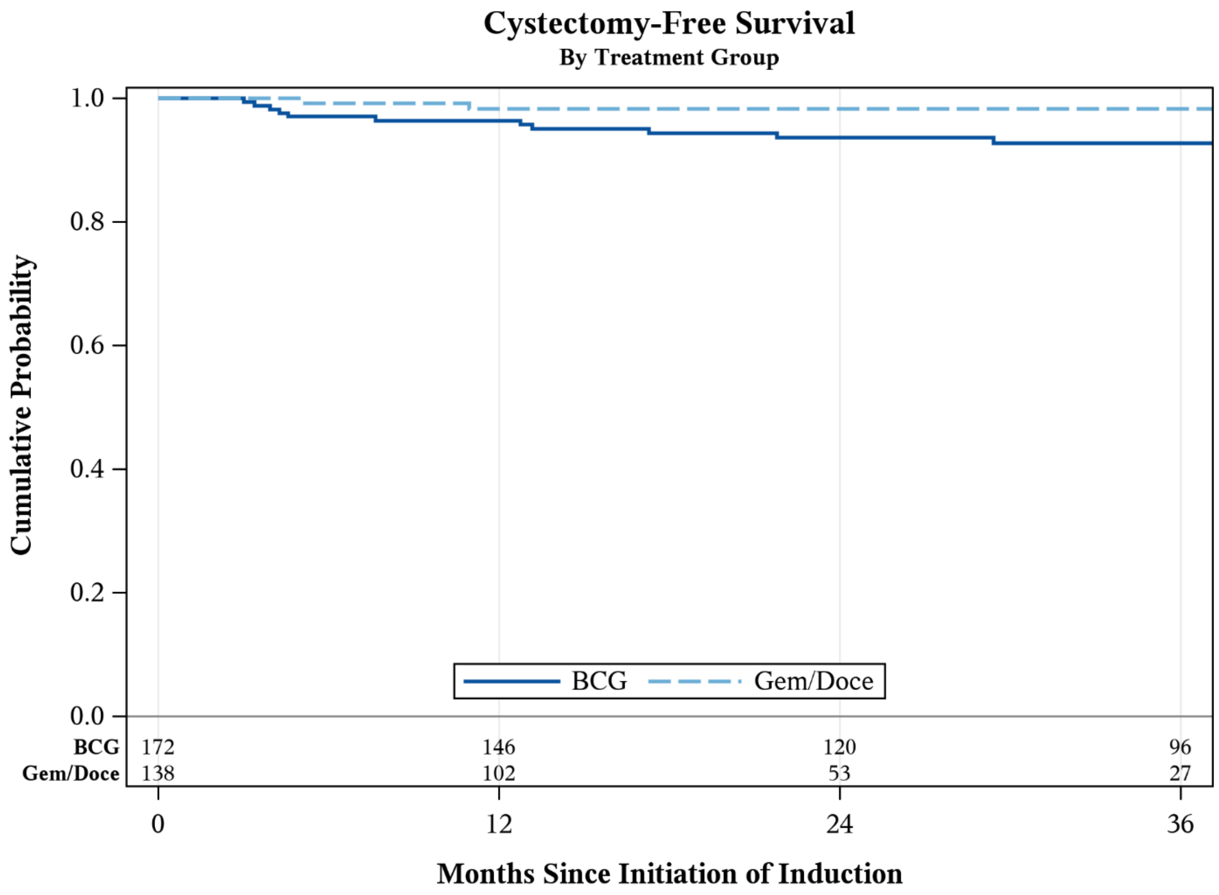

© 2023 McElree IM et al. *JAMA Network Open*.

eFigure 4. Cancer-Specific Survival by Treatment Group

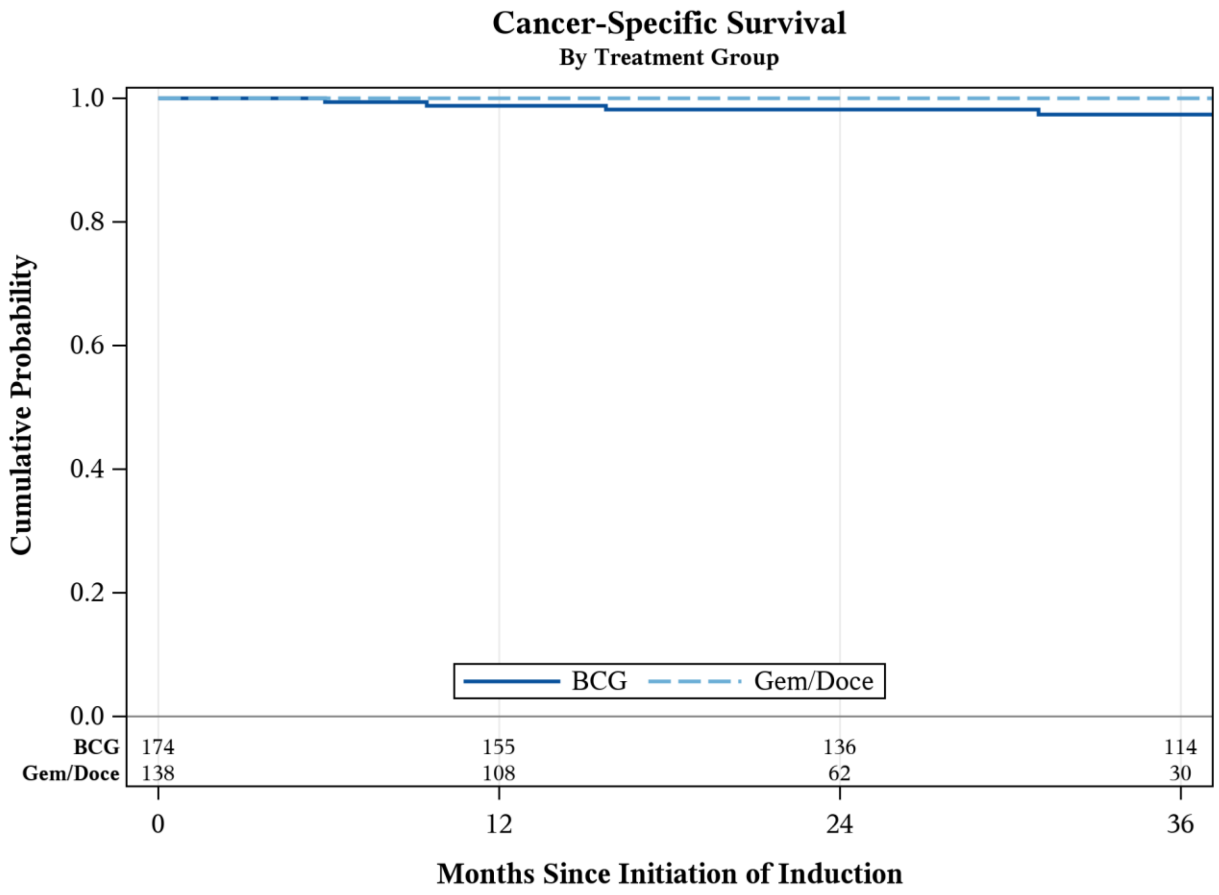

© 2023 McElree IM et al. *JAMA Network Open*.

eFigure 5. Overall Survival by Treatment Group

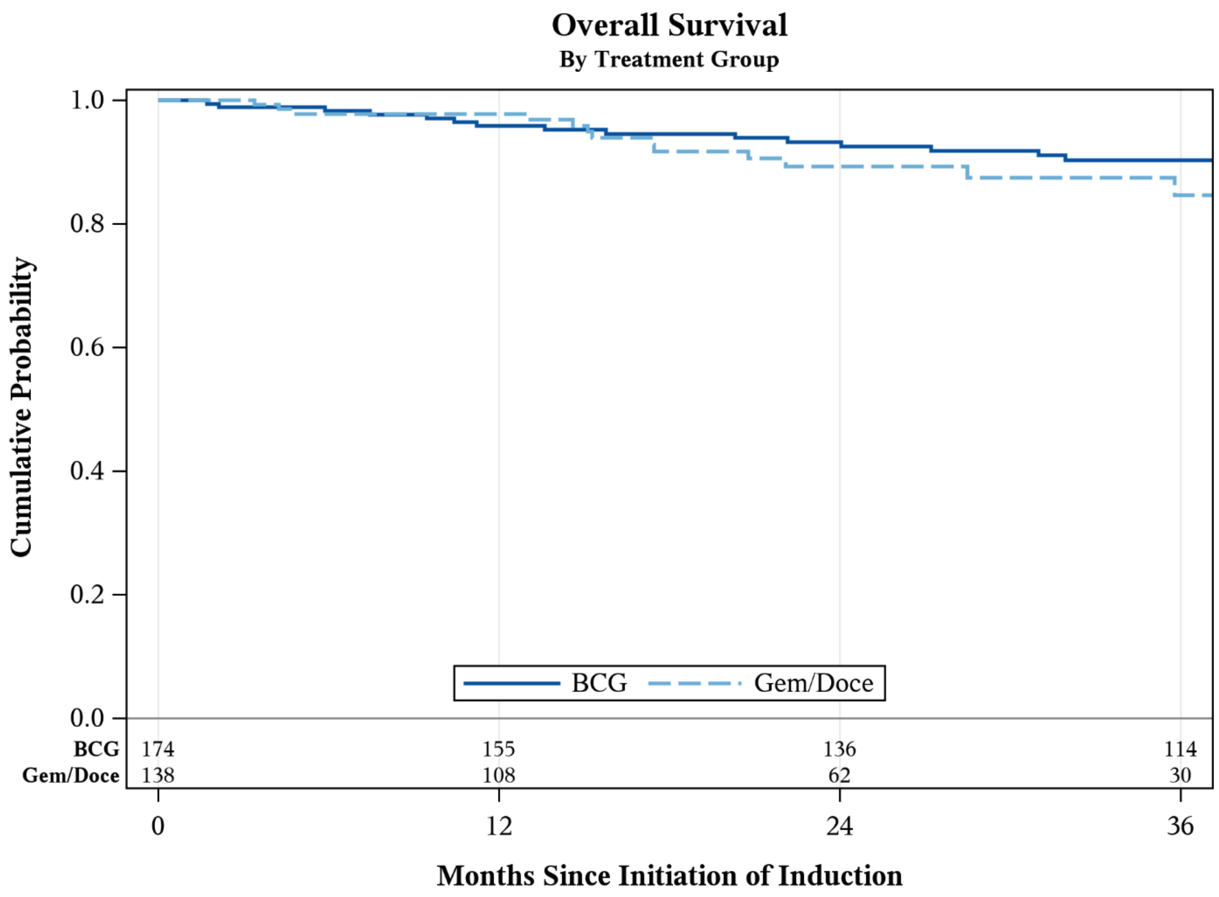

© 2023 McElree IM et al. *JAMA Network Open*.
