# Supplementary material for: Comparison of Sequential Intravesical Gemcitabine and Docetaxel vs Bacillus Calmette-Guérin for the Treatment of Patients With High-Risk Non–Muscle-Invasive Bladder Cancer
Source: JAMA Netw Open. 2023 Feb 28;6(2):e230849. doi: 10.1001/jamanetworkopen.2023.0849 (PMC9975907; doi:10.1001/jamanetworkopen.2023.0849)
Supplement: Supplement 2. — Data Sharing Statement [file jamanetwopen-e230849-s002.pdf]

Data Sharing Statement

McElree. Comparison of Sequential Intravesical Gemcitabine and Docetaxel vs Bacillus Calmette-Guérin for the Treatment of Patients With High-Risk Non-Muscle–Invasive Bladder Cancer. *JAMA Netw Open*. Published February 28, 2023.
doi:10.1001/jamanetworkopen.2023.0849

Data
Data available: No

Additional Information
Explanation for why data not available: IRB limitations of confidential patient information
